# Rare Cases of IDH1 Mutations in Spinal Cord Astrocytomas

**DOI:** 10.32607/actanaturae.10915

**Published:** 2020

**Authors:** N. A. Konovalov, D. S. Asyutin, E. G. Shayhaev, S. V. Kaprovoy, S. Yu. Timonin

**Affiliations:** National Medical Research Center of Neurosurgery, Ministry of Health of the Russian Federation Acad. N.N. Burdenko, Moscow, 125047 Russia; FGBU Russian Research Center for X-ray Radiology of the Ministry of Health of the Russian Federation, Moscow, 117485 Russia

**Keywords:** IDH1, IDH2, spinal cord astrocytoma, NGS

## Abstract

A low occurrence rate of spinal cord gliomas (4.3% of primary and glial CNS tumors) and the associated difficulties in building statistically significant cohorts of patients considerably slow down the development of effective approaches to the treatment of spinal cord tumors compared to brain tumors. Despite our extensive knowledge regarding *IDH *mutations in intracranial tumors, mutations of this gene in spinal cord astrocytomas remain poorly understood. In this study, we report on five cases of identified mutations in the *IDH1 *gene in spinal cord astrocytoma cells, two of which are unique, as they have never been previously described in CNS gliomas.

## INTRODUCTION

Brain tumors are the most common and, therefore, most studied primary tumors of the central nervous system (CNS). According to CBTRUS 2014, primary spinal cord tumors account for 4.3% of primary and glial CNS tumors; these include ependymomas (21%), astrocytomas including glioblastomas (3.2%), tumors of various nature (metastases, lymphomas, other neuroepidermal tumors) (5.9%), and piloid astrocytomas (0.8%). The low occurrence rate of spinal cord gliomas and the associated difficulties in building statistically significant cohorts of patients significantly slow down the study of the mechanisms of emergence, progression, and development of approaches to an effective treatment of spinal cord gliomas compared to brain tumors.

To date, the investigation of intracranial astrocytomas has identified a number of genetic markers that enable us to grade the malignancy of these tumors, predict the course of the disease, and, in some cases, facilitate targeted therapy.

One of the most important findings in the investigation of the cells of brain gliomas (including astrocytomas) was the identification of somatic missense mutations in the *IDH1 *and *IDH2 *genes encoding isocytrate dehydrogenase 1 and 2. *IDH1/2 *mutations are most often associated with grade II–III astrocytomas and secondary glioblastomas (70–80% of cases). They are rather rarely ( < 5%) detected in pilocytic astrocytomas and primary glioblastomas, which makes them good diagnostic markers [1]. In addition, they serve as an important prognostic indicator: anaplastic astrocytomas with wild-type *IDH *are more aggressive and closer to glioblastomas than tumors with *IDH *mutations; the most favorable course of the disease is typical of a combination of an *IDH *mutation and 1p/19q codeletion [2]. Finally, this biomarker may also be potentially useful in the development of drugs for targeted tumor therapy. For example, study [3] revealed the partial effectiveness of selective isocitrate dehydrogenase inhibitors in tumors with the R132H *IDH1 *mutation both *in vitro* and in glioma models. The mentioned characteristics of *IDH *mutations became the reason for their official inclusion in the list of biomarkers for the classification of CNS tumors according to WHO criteria [2].

**Table:** IDH1 mutations identified in the study

Mutation^*^	Intramedullary tumor, localization	Histological diagnosis	Age and gender of the patient
R132H	Two tumor nodes at the C3–C5 and T2–T3 levels	Piloid astrocytoma	F, 32
R132H	C2–C3	Anaplastic astrocytoma	F, 28
R132G	C5–C7	Diffuse astrocytoma	F, 33
R82K	C7–T1	Piloid astrocytoma	F, 21
I76T	C1–C6	Piloid astrocytoma	M, 19

^*^R – arginine,

H – histidine,

G – glycine,

K – lysine,

I – isoleucine,

T – threonine.

In glioma cells, the most often heterozygous single nucleotide substitution in the *IDH1 *gene leads to the replacement of an arginine residue with histidine at position 132 (R132H, > 90% of cases) in the enzyme active center. Substitutions of arginine by cysteine, serine, glycine, leucine, valine, and proline are much less common [4]. There are also reports of dinucleo tide insertions/deletions leading to the replacement of arginine-132 with cysteine (two cases of anaplastic astrocytoma and one case of brain glioblastoma) [5] and valine (one case) [6], as well as two cases of homozygous mutations in anaplastic astrocytoma cells – replacement of arginine-132 with leucine [5] and histidine [7]. There are reports of a rare substitution of arginine by glutamine at position 100 (R100Q) in isocitrate dehydrogenase, which also leads to a loss of the function of the protein: two cases of this substitution in anaplastic oligodendroglioma and one in diffuse astrocytomas [8]. One case of the R100Q mutation was detected in glioblastoma cells [5].

Despite our extensive knowledge about the mutations in the *IDH *gene in intracranial tumors, information on mutations in spinal cord astrocytomas (SCAs) is extremely limited, which is associated with the rarity of these tumors and, therefore, with a too-small size of the samples to enable a reliable statistical analysis. To date, we have unearthed information about two cases of an *IDH1 *mutation in diffuse SCA cells and one report of an *IDH2 *mutation in pilocytic SCA cells [9, 10]. However, the differences in the mechanisms of emergence and development of CNS tumors of different localizations complicate any extrapolation of data on brain tumors to spinal tumors. Therefore, the need for an accumulation and analysis of information on *IDH* mutations in SCA cells remains very actual.

In this study, we report on five mutations in the* IDH1 *gene identified in SCA cells. Two of these mutations may be considered unique, because they are first described in CNS gliomas.

## EXPERIMENTAL

Our study of genetic mutations included 50 patients with intramedullary gliomas of the spinal cord; however, in this paper, we focused on 5 patients. Clinical manifestations were typical of the disease and included reduced sensitivity and weakness in the extremities. Patients underwent microsurgical removal of the intramedullary tumor under neurophysiological monitoring. The outcome of surgical treatment was satisfactory in all cases.

**DNA isolation**

DNA was isolated from formalin-fixed and paraffin- embedded (FFPE) tumor tissue samples using a commercial GeneRead DNA FFPE kit (QIAGEN, USA). DNA suitable for further analysis was collected; the DNA concentration varied from 10 to 100 ng/μL.

**Next generation sequencing (NGS)**

We analyzed published results of CNS tumor genetics and selected 15 genes (*ATRX*, *EGFR*, *FGFR2*, *H3F3A*,* IDH1*, *IDH2*, *NF1*, *NF2*, *NTRK1*, *PDGFRA*, *PIK3CA*,* PIK3R1*, *PTEN*, *PTPN13*, *TP53*) whose mutations may be related to the molecular pathogenesis of spinal cord and brain astrocytomas. On the basis of the AmpliSeq technology, with the participation of the Illumina company, a primer panel was developed, which enabled a selective analysis of these genes in our DNA samples on a MiSeq next-generation sequencer (Illumina, USA).

**Analysis of NGS results**

Alignment of the read sequences to the reference human genome sequence (hg19) and their quality filtration were performed automatically using the MiSeq Reporter software (Illumina). Analysis (annotation) of genetic changes was conducted using the Variant Studio 3.0 software (Illumina). The Integrated Genomics Viewer (IGV) software (Broad Institute, USA) was used to visualize genomic data, evaluate the read depth, and identify possible false-positive results. The population rates of annotated genetic variants were evaluated using data from the gnomAD project (gnomad. broadinstitute.org/variant/2-209113262-C-T?dataset=gnomad_r2_1). The influence of genetic variants of genes on the structure and functions of encoded proteins was analyzed using the Pathoman (pathoman. mskcc.org/PathoMANmethodDescription) and Condel (bbglab.irbbarcelona.org/fannsdb/) bioinformatics methods. Their clinical significance was assessed using the NCBI ClinVar database (ncbi.nlm.nih.gov/clinvar/).

## RESULTS AND DISCUSSION

No mutations in the *IDH2 *gene were found in any of the 46 analyzed samples. However, mutations in the* IDH1 *gene were identified in five samples (*Table*).

Because mutations in the *IDH1 *gene in spinal cord astrocytoma cells have been described only in two publications [9, 10] to date, with one of them lacking mutation details, these results have a high degree of novelty. In the *IDH1 *gene isolated from diffuse spinal cord astrocytoma, a mutation underlying the R132S amino acid substitution (Arg→Ser) was detected in the enzyme molecule [9]. Our results demonstrate that two more substitutions may occur in SCA cells at this IDH1 position: R132H (Arg→His) and R132G (Arg→Gly). Both of these substitutions were found in *IDH1 *in brain astrocytomas, with the first of them being the most common variant.

**Fig. 1:**
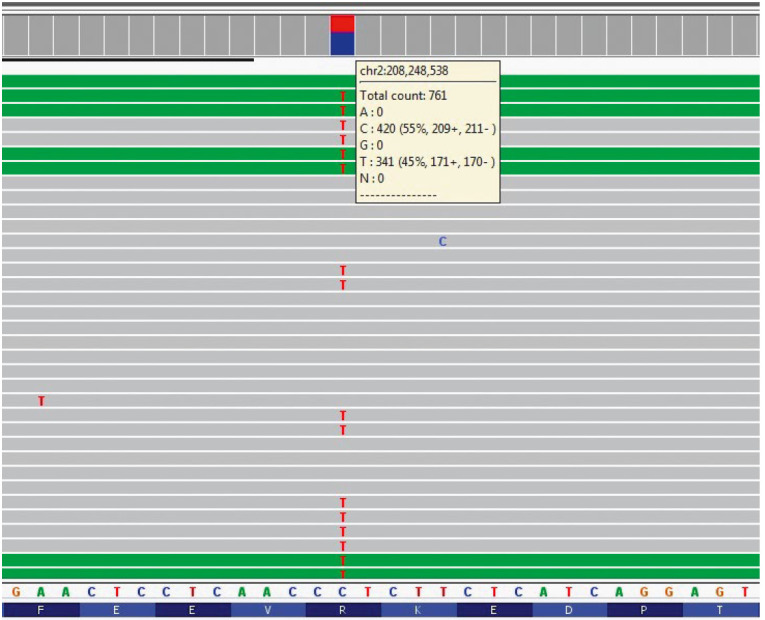
Identification of the c.245G> A mutation (p.R82K) in the *IDH1 *gene by NGS. A diagram of multiple reads of a chromosome 2 fragment involving the *IDH1 *gene (Integrative Genomics Viewer (IGV)) is shown. The C> T substitution at position 208248538 of chromosome 2, which corresponds to the c.245G> A substitution in the *IDH1* gene, is shown in red. The total read depth of this region is 761X; the number of reads of the mutant nucleotide T is 341 (45%)

**Fig. 2:**
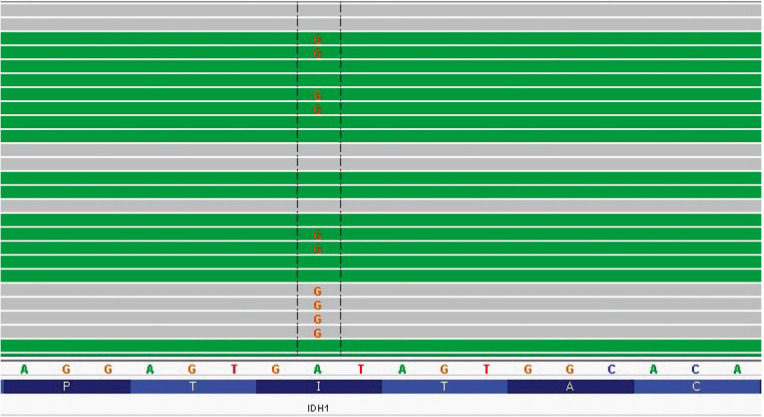
Identification of the c.227T> C mutation (p.Ile- 76Thr) in the *IDH1 *gene by NGS. A diagram of multiple reads of a chromosome 2 fragment involving the *IDH1* gene (Integrative Genomics Viewer (IGV)) is shown. The A> G substitution at position 208248556 of chromosome 2, which corresponds to the c.227T> C substitution in the* IDH1 *gene, is shown. The total read depth of this region is 585X, and the number of reads of the mutant nucleotide G is 317 (54%)

Of particular interest are two more substitutions at positions 82 (Arg→Lys, R82K) and 76 (Ile→Thr, I76T) of IDH1 (*Fig. 1*, *Fig. 2*). These are hereditary mutations with very low occurrence rates. According to the gnomAD resource, the population rate is 0.002475% for the R82K variant and 0.0003977% for the I76T variant. Both of these substitutions are described for the first time not only in spinal cord astrocytomas, but also in CNS tumors in general. We were able to find only two publications where the first mutation (R82K) was described in patients with acute myeloid leukemia [11] and primary skin melanoma [12]. We did not find data on the second mutation (I76T). It is worth noting that the samples with these unique mutations did not contain any of the other astrocytoma-associated mutations included in the analysis panel (*ATRX*, *EGFR*, *FGFR2*, *H3F3A*,* NF1*, *NF2*, *NTRK1*, *PDGFRA*, *PIK3CA*, *PIK3R1*, *PTEN*,* PTPN13*, *TP53*; data not shown).

Our methods for predicting the effect of the identified amino acid substitutions on the structure and function of the proteins (Pathoman and Condel) point to a damaging effect of the R82K and I76T substitutions. In the NCBI ClinVar database, these mutations are classified as variants with unknown significance. More detailed research is needed to determine their possible effect.

Therefore, our study unearthed new information on the mutations in the *IDH1 *gene in SCA cells. We found two unique mutations that had not been previously described in the cells of central nervous system tumors. The additional significance of the obtained results is associated with the fact that the WHO official classification of tumors envisages the use of *IDH1 *mutations as one of the markers with a high diagnostic and prognostic value. Because the R132H variant accounts for most of the mutations in this gene, immunohistochemical staining with appropriate antibodies is usually used for any analysis. However, identification of other mutant variants in this case becomes impossible. In our case, of the five identified mutations, only two belonged to the dominant variant; in addition, they happened to be unique. Therefore, these mutations can be identified only by sequencing. In this regard, NGS sequencing should be used to identify rare mutations in the *IDH* genes in SCA samples.

All study procedures involving human participants complied with the ethical standards of the institutional and/or national committee for research ethics and with the 1964 Helsinki Declaration and its later amendments or comparable ethical standards.

Informed voluntary consent was obtained from all participants enrolled in the study.

## Abbreviations

CNS: central nervous system
IGV: Integrative Genomics Viewer
FFPE: formalin-fixed paraffin-embedded
SCA: spinal cord astrocytoma
